# Development of a short course on management of critically ill patients with acute respiratory infection and impact on clinician knowledge in resource‐limited intensive care units

**DOI:** 10.1111/irv.12569

**Published:** 2018-05-26

**Authors:** Janet V. Diaz, Justin R. Ortiz, Paula Lister, Nahoko Shindo, Neill K.J. Adhikari, WHO Critical Care Training Short Course collaborators

**Affiliations:** ^1^ Infectious Hazard Management Health Emergency Programme World Health Organization Geneva 27 Switzerland; ^2^ Institute for Global Health University of Maryland School of Medicine Baltimore MD USA; ^3^ Paediatric Critical Care Unit Sunshine Coast University Hospital Birtinya Qld Australia; ^4^ Department of Critical Care Medicine Sunnybrook Health Sciences Centre and University of Toronto Toronto ON Canada

**Keywords:** acute respiratory distress syndrome, acute respiratory infection, education, influenza, low‐ and middle‐income countries, sepsis

## Abstract

**Background:**

The 2009 influenza A (H1N1) pandemic caused surges of patients in intensive care units (ICUs) in resource‐limited settings. Several Ministries of Health requested clinical management guidance from the World Health Organization (WHO), which had not previously developed guidance regarding critically ill patients.

**Objective:**

To assess the acceptability and impact on knowledge of a short course about the management of critically ill patients with acute respiratory infections complicated by sepsis or acute respiratory distress syndrome delivered to clinicians in resource‐limited ICUs.

**Methods:**

Over 4 years (2009‐2013), WHO led the development, piloting, implementation and preliminary evaluation of a 3‐day course that emphasized patient management based on evidence‐based guidelines and used interactive adult‐learner teaching methodology. International content experts (n = 35) and instructional designers contributed to development. We assessed participants’ satisfaction and content knowledge before and after the course.

**Results:**

The course was piloted among clinicians in Trinidad and Tobago (n = 29), Indonesia (n = 38) and Vietnam (n = 86); feedback from these courses contributed to the final version. In 2013, inaugural national courses were delivered in Tajikistan (n = 28), Uzbekistan (n = 39) and Azerbaijan (n = 30). Participants rated the course highly and demonstrated increased immediate content knowledge after (vs before) course completion (*P* < .001).

**Conclusions:**

We found that it was feasible to create and deliver a focused critical care short course to clinicians in low‐ and middle‐income countries. Collaboration between WHO, clinical experts, instructional designers, Ministries of Health and local clinician‐leaders facilitated course delivery. Future work should assess its impact on longer‐term knowledge retention and on processes and outcomes of care.

## 1 INTRODUCTION

In low‐ and middle‐income countries (LMICs), as defined and classified by the World Bank on the basis of gross national income per capita,^1^ the burden of critical illness is high, with a significant proportion attributable to acute infections.^2^ In particular, lower respiratory infections are the third leading cause of disability‐adjusted life years lost and disproportionately affect Africa and the Eastern Mediterranean and South East Asian regions.^3^ Thus, improving the care of critically ill patients with acute respiratory infections is of public health importance.

A major challenge to meeting this objective is the global variation in the quantity and quality of critical care delivery.^2^, ^4^ LMICs may have limited access to basic hospital infrastructure,^5^ essential health technologies (oxygen, medicines),^6^ intensive care units (ICUs) and other resources (including evidence‐based practice protocols).^5^, ^7^, ^8^, ^9^, ^10^, ^11^ Even in ICUs with life support technologies, healthcare staff may not be adequately trained.^12^

The need to strengthen healthcare systems to improve care for patients with acute respiratory infections was emphasized by the global experience with severe acute respiratory syndrome (SARS),^13^ avian influenza A (H5N1),^14^ the 2009 influenza A (H1N1) pandemic,^15^, ^16^ Middle East respiratory syndrome coronavirus (MERS‐CoV) 17, 18 and avian influenza A (H7N9).^19^, ^20^ Many patients with these emerging pathogens developed respiratory failure or other organ dysfunction, creating a surge of critically ill hospital admissions in constrained healthcare systems. Therefore, outbreaks of infectious diseases causing critical illness could potentially be detected in hospital settings that care for such patients, such as the ICU and emergency department, which therefore become logical targets for health system strengthening.

During the 2003 SARS epidemic, the WHO formed a core clinical advisory network. In response to human infections with avian influenza A (H5N1) and the 2009 influenza A (H1N1) pandemic, this network produced clinical management guidance,^21^, ^22^ consolidated knowledge into review articles^23^, ^24^ and joined outbreak investigations. Most recently, and in a novel development for the WHO, this network created an evidence‐based critical care short course to assist physicians and nurses in resource‐limited ICUs, many without formal critical care training, to care for critically ill patients with acute respiratory infections. The objective of this report was to assess the acceptability to clinicians and impact on their knowledge of this course. Preliminary results were presented in abstract form.^25^

## 2 METHODS

### 2.1 Overview

Phases in the development of the Critical Care Training Short Course are described in Table 1. In both the pilot and implementation phases, we assessed participants’ satisfaction (using self‐administered surveys) and content knowledge (using a written multiple‐choice test) before and after the course.

**Table 1:** Development timeline for Critical Care Training Short Course

Date	Activities
2003	WHO creates a global clinical network during the SARS epidemic, which expands during the avian influenza A (H5N1) outbreaks and 2009 influenza A (H1N1) pandemic
	Network activities develop global clinical management guidance consolidate knowledge into peer‐reviewed review articles investigate outbreaks conduct clinical research and training sessions
2‐4 December 2009	WHO convenes meeting of the “Workgroup on Pandemic (H1N1) Critical Care Training Module Development” in Geneva, Switzerland to develop training materials in response to member states’ requests for assistance
	Meeting output: 13 peer‐reviewed computer‐based presentations
20‐21 October 2010	WHO convenes meeting on the “Clinical Management of Influenza and Other Acute Respiratory Illness in Resource‐Limited Settings: Learning from the Influenza Pandemic (H1N1) 2009” in Geneva, Switzerland
	Based on feedback, a 3‐day, adult‐learner friendly, short course is created with 14 learning sequences, short lectures with animations, interactive role plays for small group sessions, and a clinical management toolkit
5‐7 April 2011	Pilot #1. Sub‐regional Workshop on Critical Management of Respiratory Diseases, Port of Spain, Trinidad and Tobago
30 April‐3 May 2012	Pilot #2. Workshop on Critical Management of Severe Acute Respiratory Infections, Bogor, Indonesia
7‐9, 13‐15 May 2013	Pilot # 3: Clinical Management of Severe Influenza Infections: short course, Hanoi and Ho Chi Minh City, Vietnam
June 2013	Materials revised based on new published evidence
	Pilot experiences integrated
July‐October 2013	Short course materials published
	Course delivered in Uzbekistan, Azerbaijan, and Tajikistan (national implementations)

SARS, severe acute respiratory syndrome.

We did not seek research ethics board approval because this course and its evaluation were designed and conducted as part of routine WHO activities.

### 2.2 Preparation

#### 2.2.1 Call to action

During the 2009 influenza A (H1N1) pandemic, the WHO received requests for clinical management advice from several Member States classified as LMICs. A review within the WHO and the WHO clinical network on severe influenza identified useful clinical materials but none that provided adequate patient management advice. Therefore, WHO decided to create critical care training materials that could be deployed during public health emergencies. These materials were designed to assist physicians and nurses working in ICUs in LMICs to care for patients with acute respiratory infections.

#### 2.2.2 Development of core content of training materials

WHO convened a “Workgroup on Pandemic (H1N1) Critical Care Training Module Development,” which met 2‐4 December 2009 in Geneva, Switzerland, and was comprised of twelve experts in critical care, virology, obstetrics and public health to develop the technical basis of the course. All experts were from academic or public health institutions and had experience working in LMICs.

This WHO workgroup developed teaching materials based on the following assumptions: (i) participants would have basic knowledge of hospital‐based acute care medicine, including airway management and initial set‐up of mechanical ventilation (eg, from anaesthesia or emergency department practice), (ii) participants’ clinical environment (district or tertiary hospitals) would include ICUs able to provide oxygen, mechanical ventilation and vasoactive medication infusions, (iii) the course would focus on the syndromic management of acute respiratory distress syndrome (ARDS) and sepsis, 2 complications of acute respiratory infection, including from pandemic influenza A (H1N1) virus. We also assumed that many participants would have no formal critical care training, but that in some countries, intensivists might exist and be interested in attending the course.

Workgroup members identified eleven topics encompassing common issues related to critical care of acute respiratory infection, including severe influenza infection (Table 2). For each topic, a primary author was assigned to create a 1‐hour lecture using a computer‐based presentation (Powerpoint, Microsoft, Redmond, WA, USA) with an instructor script, which was then reviewed by 3 or 4 experts. Two computer‐based presentations included case studies.

**Table 2:** Technical content of Critical Care Training Short Course: draft and final versions

Unit number	Original draft materials, December 2009	Final materials, April 2011
1	Influenza basics	Introduction to critical care management of severe influenza infection
2	Clinical basics	Diagnose severe forms of influenza infection
3	Diagnostics and specimen collection	Deliver oxygen therapy
4	Antimicrobial therapy	Differential diagnosis and diagnostic tests
5	Hospital infection control	Deliver targeted resuscitation to patients with sepsis
6	Clinical management on the hospital wards	Monitor the patient
7	Approach to patient in septic shock	Antimicrobial therapy and its modification after influenza test interpretation
8	Approach to ARDS	Deliver lung protective mechanical ventilation to patients with acute respiratory distress syndrome
9	Best practice for ICU management of severely ill patient	Deliver targeted sedation and prevent delirium
10	Paediatric Influenza	Formulate a treatment plan to prevent complications
11	Influenza in pregnancy	Liberate patients from mechanical ventilation using spontaneous breathing trial
12	Case exercise #1	Deliver quality critical care services
13	Case exercise #2	Implement measures to prevent and control infection when caring for patients with acute respiratory infection
14	‐	Apply ethical principles in decision‐making

#### 2.2.3 Peer‐review process

Over the next few months, 35 international experts (Appendix S1) contributed feedback or materials to the course. Experts had specialty training in 1 or more of critical care, pulmonary disease, infectious disease, paediatrics, obstetrics, epidemiology, anaesthesia, radiology and clinical pharmacy. They represented eleven countries: Australia, Canada, Dominican Republic, Hong Kong Special Administrative Region, India, Japan, Mexico, Nepal, South Africa, United Kingdom and United States of America. All authors and peer‐reviewers completed WHO conflict of interest declarations; none had a conflict of interest that precluded participation in the course development process.

The draft Critical Care Training Short Course was presented during the WHO convened meeting “Clinical Management of Influenza and Other Acute Respiratory Illness in Resource‐Limited Settings: Learning from the Influenza Pandemic (H1N1) 2009” in October 2010.^24^ More than 100 clinicians, public health practitioners and scientists shared their experiences, of whom 30 provided detailed feedback on the computer‐based presentations. Participants reported the technical content to be pertinent and up to date, but recommended that the presentations be transformed into a more interactive, practical and adult‐learner friendly format.

#### 2.2.4 Transformation of training materials into 3‐day short course

In response, the WHO partnered with Agence de Médecine Préventive (a non‐profit organization for preventive medicine and public health with instructional design expertise, Paris, France) to transform the materials into a 3‐day integrated and interactive training course with fourteen learning units (Table 2) that employed adult‐learner training methodology. The following changes were made to the original draft materials:

1. Content pertaining to the care of children and pregnant women was integrated into the other learning units, highlighting differences when appropriate.
2. The lectures were reorganized into learning units aligned with the timing of clinical actions. Each revised learning unit had 3‐5 learning objectives and included a computer‐based presentation (15‐30 slides) and a small group interactive session to reinforce learning points (maximum, 8 participants with 1 instructor).
3. Case scenarios were created (4 adults, including a pregnant woman; 1 infant) for small group interactive sessions to facilitate problem‐based learning and reinforce learning objectives.
4. Animations embedded into appropriate lectures were created to present selected physiological concepts (i.e hypoxaemia, ARDS, septic shock and lung protective ventilation).
5. A clinical toolkit was created with treatment guidelines, checklists, fact sheets, algorithms, information tables and references suitable for the small group sessions and subsequent clinical practice.
6. Two learning units were added on quality improvement and clinical ethics.

All training materials were developed in English and were packaged on a CD with computer‐based presentations, animations, role‐play cards for the case scenarios, the clinical toolkit, and an instructor guide with written scripts for lectures, notes for role‐play scenarios, a sample workshop schedule, pre‐ and post‐tests and daily evaluation forms [see below] and a complementary resource folder with other pertinent WHO documents.

After each pilot course, revisions were made to materials based on feedback from participants (Table S1).

#### 2.2.5 Development of course evaluation materials

We developed a self‐administered participant satisfaction survey with questions regarding learning environment, teaching materials and methods, and facilitators. Response frames used a Likert scale (range, 1‐5, very poor to very good) with space for free‐text comments. We also developed a multiple‐choice single best answer test (25 questions; range of possible scores, 0‐25) based on core concepts. The test was administered to participants immediately before and after the course to assess change in their knowledge. The satisfaction survey and test were reviewed by 4 members of the course leadership (JVD, PL, NS and NKJA) and iteratively refined by email and after the pilot phase.

### 2.3 Data analysis

We report descriptive data as percentages (categorical data) and means and standard deviations (continuous data, which were all normally distributed). At each course, (i) mean scores on the pre‐test and post‐test were compared using unpaired t tests (because participants’ names were not consistently recorded), (ii) a mean overall satisfaction score for each participant was calculated for each of 14 learning units and for the introduction (possible range, 1‐5, with 1 denoting very poor and 5 denoting very good). For each workshop, we report the mean of these mean scores and the range of the means.

We report the results for pilot courses by country because the content was iteratively modified after each course. For the implementation in Central Asia, the courses were delivered over 4 months, with the materials unchanged, and we therefore reported results from all participants together. In addition, we report qualitative comments from course participants and facilitators.

## 3 RESULTS

### 3.1 Pilot phase: delivery and evaluation

The short course was piloted in 3 sites, Trinidad and Tobago (29 participants, April 2011), Indonesia (38 participants, May 2012) and Vietnam (86 participants, May 2013), selected based on interest from WHO Regional and Country Offices and the national Ministries of Health (Appendix S1). The Ministries of Health nominated all participants, with no direct personal costs. Although most participants were ICU clinicians, many Ministries of Health invited non‐clinical participants (eg, field epidemiologists involved with operational aspects of outbreak response) to improve communication with clinicians and help them overcome challenges (eg, timely specimen collection, transportation and processing). All courses were delivered by an international group of expert facilitators, mostly intensivists and infectious diseases physicians, over a 3‐day period after meeting 1 day in advance to review course structure and facilitation techniques and to visit local ICUs. Details of each pilot are provided in Appendix S1. Learners in all 3 pilots rated the learning units as good to very good (range of mean Likert scores 3.9 to 4.9), with significant improvement in test scores after training (*P* < .001, Table 3).

**Table 3:** Critical Care Training Short Courses: participants’ evaluation and test performance

	Pilot phase			Implementation phase
	Trinidad (April 2011)	Indonesia (May 2012)	Vietnam (May 2013)	Central Asia (August‐October 2013)
Number of participants	29	38	86	97
Range of mean ratings (introduction and 14 learning units)^a^	4.5‐4.9	3.9‐4.3	4.5‐4.6	4.6‐4.8
Pre‐test score, mean (SD), n	14.4 (4.0), n = 29	14.1 (3.0), n = 36	16.2 (3.9), n = 76	10.6 (3.9), n = 76
Post‐test score, mean (SD), n	20.0 (2.3), n = 29	19.3 (3.5), n = 37	21.2 (1.8), n = 45	14.4 (3.8), n = 87
*P* value^b^	<.001	<.001	<.001	<.001

a For the satisfaction ratings, 1 denoted very poor and 5 denoted very good.

b *P* values are from unpaired *t* tests of post‐test vs pre‐test scores (possible range of scores, 0‐25).

### 3.2 Implementation phase: delivery and evaluation

In 2013, the Critical Care Training Short Course conducted inaugural implementation courses upon request of the WHO EURO Regional Office and the Country Offices and Health Ministries of Uzbekistan, Azerbaijan and Tajikistan. This request was in response to the MERS‐CoV outbreak in Saudi Arabia and in preparation for national pilgrims attending the Haj. The computer‐based slides and toolkit were translated into Russian, and the course was facilitated in English with professional interpreters providing simultaneous translation.

In Uzbekistan, 39 participants from thirteen provinces, all senior physicians or heads of departments of referral hospitals, attended the course. In Azerbaijan, 30 physicians from 7 regions, most representing referral hospitals with a minority from district hospitals, attended the workshop. In Tajikistan, 28 physicians from twelve regions, representing national, referral and district hospitals, attended the workshop. In contrast to pilot sessions, most participants in these courses were intensive care specialists (Table S3).

Participants rated the learning units as good to very good (range of mean Likert scores 4.6‐4.8, and test scores showed significant improvement after training (*P* < .001, Table 3). On written comments, participants requested more time for the course, additional courses using a train‐the‐trainers model, and more comprehensive materials on mechanical ventilation, in addition to those provided on ARDS. Facilitators noted that many international critical care guidelines, such as those of the Surviving Sepsis Campaign, were not readily available to participants; therefore, this course introduced some core concepts.

## 4 DISCUSSION

The main findings of this work are that the development of an evidence‐based and interactive Critical Care Training Short Course delivered in 3 days to a mixed audience of 250 non‐specialty and specialty trained physicians and nurses in 6 countries was feasible, well‐received and significantly improved participants’ immediate knowledge. The course represents the first foray of WHO into critical care management and directly addresses the public health need of improving the care of critically ill patients with acute respiratory illness complicated by sepsis or ARDS in LMICs. The course is suitable for non‐specialist healthcare workers, who provide the majority of critical care in LMICs. It can be deployed rapidly to assist clinicians during public health emergencies caused by novel respiratory virus infections, but can also be incorporated into continuing medical education using a train‐the‐trainers model.

Our work has important strengths. Development of the materials was publicly financed, free of commercial influence and included input from a broad spectrum of experts with experience in resource‐limited settings. This intervention addressed the challenge of bringing evidence‐based care to the bedside of patients by adapting materials to the needs of clinicians who may not be formally trained in critical care, emphasizing the provision of safe care and using an interactive teaching approach. The teaching materials were developed in a process of peer review, refinement and pilot testing and were designed by collaboration between critical care and instructional design experts. The participants rated the course's content, structure and teaching methods highly. International facilitators delivered the course and mentored local clinicians to conduct future trainings. We also observed that the course provided a forum for local, national, regional and global networking that was valued by many clinicians.

Our approach also has limitations. First, the timeline was long, starting with initial conception in 2009 and culminating in clearance by the WHO's internal review process in 2013. Second, recommended approaches followed published guidelines where available (eg, Surviving Sepsis 26), but guidelines were often not available or were based on data from high‐income health systems. More recently, guidelines adapted for low resource settings have been published,^27^, ^28^, ^29^ but with few exceptions,^30^ the adaptation has derived from expert opinion rather than context‐specific randomized trials. Third, although experts represented ten countries, African and South East Asian countries were under‐represented, which may reflect the prominence of international academic global experts. Planned revisions will increase the representation of clinical experts in LMICs. Fourth, we did not assess the psychometric properties of the knowledge test, and short‐term knowledge retention may not predict longer‐term retention or improved patient care. Therefore, more robust evaluation is required, including with skills‐based assessments. Because we did not record participant names on each test, paired t tests were not possible, and the effect of the course on short‐term knowledge may be underestimated. Alternatively, where there was a substantial difference in number of participants between pre‐test and post‐test administrations (Vietnam, Central Asia), the improvement in scores may be an overestimate if participants with mastery were more likely to have taken the second test. Fifth, the success of this course requires acceptance by local champions and adaptation to local settings, resource constraints, and baseline knowledge and experience of participants . Finally, it is imperative to work closely with Ministries of Health to select appropriate participants.

This Critical Care Training Short Course faces multiple challenges to sustainability. The technical content must be kept up to date with evolving clinical research and guidelines. The course methodology and structure may need further revision in response to participant feedback, such as inclusion of hands‐on training with mechanical ventilators. Achieving global impact will require scaling up to reach more clinicians; recruitment, coordination and retention of expert clinician facilitators; development of a train‐the‐trainers model to sustain instruction by local clinicians; translation of materials into local languages; and development of alternate online or smartphone dissemination methods. Our experiences in Central Asia, Indonesia and Vietnam suggest that these barriers can be overcome, as these countries are planning national course implementations (Janet Diaz, Selamet Hidayat, Vu Quoc Dat; personal communications). Finally, evaluation of the course's impact on longer‐term knowledge retention, processes of care and patient‐centred outcomes is necessary. The WHO Integrated Management of Childhood Illness programme provides an example of both the benefits^31^, ^32^ and challenges^33^ of implementing teaching materials to improve clinical outcomes for seriously ill children.

Following the trainings described in this manuscript (2009‐2013), this course has been successfully delivered in China, Egypt, Palestine, Fiji, Uzbekistan, Armenia, Philippines, Tajikistan, Kyrgyzstan and Turkmenistan, with over 1000 clinicians trained. Our experience suggests that 3 days is the minimum time to deliver the content and conduct case‐based discussions to consolidate learning points without overwhelming participants with information. In countries where simultaneous translation is used, 3 days may not be enough.

## 5 CONCLUSIONS

Recent outbreaks of respiratory virus infections highlight the need for educational interventions to help clinicians to care for critically ill patients. We have shown the feasibility of creating and delivering an evidence‐based and interactive Critical Care Training Short Course for non‐specialty and specialty clinicians in resource‐limited settings, using a collaborative approach between the WHO, a global network of experts in critical care and infectious disease, instructional designers, national Ministries of Health and local clinicians. Despite challenges related to sustainability and dissemination, we believe that targeted clinical short courses integrated into national capacity‐strengthening initiatives will help healthcare systems to better care for critically ill patients with acute respiratory infections.

## AUTHORS' CONTRIBUTIONS

JVD, JRO, PL, NS and NKJA contributed to the development of the short course. JVD, PL, NS and NKJA revised the materials. JVD, PL and NKJA taught the course. JVD and NKJA conceived and designed the study and collected and analysed data. JVD and NKJA drafted the manuscript. All authors revised the manuscript and approved the final version. NKJA drafted the revised manuscript.
