# Supplementary material for: Development of a short course on management of critically ill patients with acute respiratory infection and impact on clinician knowledge in resource‐limited intensive care units
Source: Influenza Other Respir Viruses. 2018 May 26;12(5):649–55. doi: 10.1111/irv.12569 (PMC6086848; doi:10.1111/irv.12569)
Supplement: Supplementary file 1 [file IRV-12-649-s001.pdf]

Appendix S1

WHO Critical Care Training Short Course collaborators

2009-2010 original contributors and peer reviewers

Neill KJ Adhikari, Andre Amaral, Edgar Bautista, Yolanda Bayugo, Satish Bhagwanjee, Niranjan Bhat, Cheryl Cohen, Hillary Cohen, Shelly Dev, Wes Ely, Charles D Gomersall, Carlos G Grijalva, Wendy Hansen, Sabine Heinrich, Michael Ison, Shevin Jacob, Arjun Karki, Paula Lister, John Luce, Kirsten Lunghi, Shabir Madhi, Christine Olson, Justin Ortiz, Kishore Pichamuthu, Kevin Rooney, Gordon Rubenfeld, Harry Shulman, Daisuke Tamura, Monica Thormann, Tim Uyeki, Khai Vu, Eric Walter, Steven Webb, T. Eoin West, Jenson Wong.

These 35 contributors and reviewers included 17 adult intensivists, 3 paediatric intensivists, 5 infectious diseases specialists, 4 public health specialists, 2 obstetricians, 2 radiologists, 1 emergency medicine specialist, and 1 clinical pharmacist. Most were based in USA (18), with the remainder from Canada (4), South Africa (2), UK (2), Australia (1), Dominican Republic (1), Germany (1), Hong Kong SAR (1), India (1), Japan (1), Mexico (1), Nepal (1), and Switzerland (1).

2009-2013 Course facilitators

Trinidad and Tobago: Neill KJ Adhikari, Janet Diaz (external); Antony Parkinson (local)
Indonesia: Neill KJ Adhikari, Janet V Diaz, Paula Lister (external); Rudy Manalu, Dwi Pantja, Ruppi, Subdit RS Privat, Bambang Wahjuprajitno (local)
Vietnam: Neill KJ Adhikari, Janet V Diaz, Paula Lister, Rudy Manalu, Kevin Patel (external); Phan Huu Phuc, Dat Quoc Vu, Nguyen Kim Thu, Ta Thi Dieu Ngan, Than Manh Hung, Tran Van Bac (local)
Azerbaijan: Janet V Diaz, Paula Lister, Kobus Preller, Natalia Pshenichnaya, (external); Irada Akhundova (local)
Tajikistan: Janet V Diaz, Paula Lister, Michael Nekludov, Natalia Pshenichnaya, (external)
Uzbekistan: Janet V Diaz, Paula Lister, Kobus Preller, Natalia Pshenichnaya, Malcolm Semple (external); Anna Pashalishvili (local)

Collaborating partner organizations

Oloan E Tampubolon (Chief, Perhimpunan Dokter Intensive Care Indonesi [PERDICI], Jakarta, Indonesia; Nguyen Van Kinh (Director, National Hospital of Tropical Disease, Ha Noi, Vietnam); Research Institute of Lung Disease, Baku

Supplementary Results: Details of Pilot Trainings

The first pilot, a sub-regional Workshop on Critical Management of Respiratory Diseases in Trinidad and Tobago, 5-7 April 2011, was sponsored by the Pan American Health Organization and the Ministries of Health of eleven Caribbean countries (Trinidad and Tobago, Anguilla, Grenada, Belize, Jamaica, Bahamas, St. Vincent and the Grenadines, Antigua, St. Lucia, and Dominica). Twenty-nine participants (physicians and nurses) attended the course (Table S2) from these countries.

The second pilot was the Workshop on Critical Management of Severe Acute Respiratory Infections in Bogor, Indonesia, 30 April–3 May 2012 and was sponsored by the Indonesian Ministry of Health and the Perhimpunan Dokter Intensive Care Indonesia (Indonesian Intensive Care Society, PERDICI). Indonesia had been identified as a priority country to receive this training because of the numerous fatal human cases of avian influenza A [1]. Thirty-eight participants from ten Indonesian provinces attended the course (Table S2). They were approximately evenly divided between doctors and nurses. Most (n=24, 63.2%) worked at public referral hospitals for avian influenza A.

The third pilot consisted of Short Courses on Clinical Management of Severe Influenza Virus Infection, were conducted in Hanoi and Ho Chi Minh City, Vietnam, 7-9 and 13-15 May 2013, sponsored by the Vietnamese Ministry of Health and the National Hospital of Tropical Disease (Hanoi). Vietnam had been identified as a priority country to receive this training because of the high numbers of fatal human cases of avian influenza A (H5N1) [2, 3]. Course materials (computer-based slides and toolkit) were translated into Vietnamese. The course was delivered in English, with simultaneous interpretation. Eighty-six participants from 49 provinces, all doctors, representing provincial and national hospitals, attended the course (Table S2).

Table S1. Comments from participants in pilot short courses and iterative changes made

	Participant comments by category	Changes made
1	Expand learning sequences on lung protective ventilation for ARDS, because local knowledge regarding safe mechanical ventilation is weak. Adapt for resource-limited settings (e.g. delivery of lung-protective ventilation without arterial blood gas analysis). Include more guidance for clinicians working in ICUs without arterial blood gas analyzers.	Learning sequence on 'Invasive mechanical ventilation for ARDS' expanded to include<br>--more basics on mechanical ventilation<br>--information about diagnosis of ARDS using SaO_2/FiO_2 ratio<br>--use of end-tidal CO_2 monitoring of ventilation
2	Include hands-on sessions with a mechanical ventilator or simulator	Hands-on session added to learning sequence on 'Invasive mechanical ventilation for ARDS', subject to local availability of ventilators
3	Include more paediatric content.	Each lecture includes enhanced information about pediatrics
4	Translate materials into the national language	Materials have been translated into local languages, including Vietnamese, Russian, Bahasa, and Mandarin
5	Clarify some test questions found to be confusing	New test questions were created

Table S2. Characteristics of participants attending pilot short courses

Education	Trinidad and Tobago, April 2011 (n=29)	Indonesia, May 2012 (n=38)	Vietnam, May 2013 (n=86)
Doctor	17 (58.6%)	18 (47.4%)	86 (100%)
Intensive Care			
Anesthesiology	5	5	0
Internal medicine	3	0	0
Pulmonary disease	1	2	0
Paediatrics	3	0	0
General practice	3	0	0
Emergency medicine	0	0	69
Infectious disease	0	0	27
Medical Epidemiologist			
Trainee	2	0	0
Unknown	--	11	--
Nurse	8 (27.6%)	20 (52.6%)	0
Bachelors	--	9	--
Diploma	--	11	--
Unknown	8	--	--
Medical administrator	1 (3.4%)	0	0
Medical officer	3 (10.3%)	0	0

--, not recorded or not applicable.

Table S3. Characteristics of participants attending implementation courses in Central Asia

Education	Uzbekistan, August 2013 (n=39)	Azerbaijan, September 2013 (n=30)	Tajikistan, October 2013 (n=28)
Doctor	39 (100%)	30 (100%)	28 (100%)
Intensive Care	31	20	18
Anesthesiology	1		
Pulmonary disease		5	2
Paediatrics	3		
General practice	1		
Infectious disease	2		8
Medical Epidemiologist	1	5	

References

1. Daniels P, Wiyono A, Sawitri E, Poermadjaja B, Sims LD: **H5N1 Highly Pathogenic Avian Influenza in Indonesia: Retrospective Considerations**. *Curr Top Microbiol Immunol* 2012 Sep 6, **365**:171-184.
2. Tran TH, Nguyen TL, Nguyen TD, Luong TS, Pham PM, Nguyen v V, Pham TS, Vo CD, Le TQ, Ngo TT *et al*: **Avian influenza A (H5N1) in 10 patients in Vietnam**. *N Engl J Med* 2004, **350**(12):1179-1188.
3. Kawachi S, Luong ST, Shigematsu M, Furuya H, Phung TT, Phan PH, Nunoi H, Nguyen LT, Suzuki K: **Risk parameters of fulminant acute respiratory distress syndrome and avian influenza (H5N1) infection in Vietnamese children**. *J Infect Dis* 2009, **200**(4):510-515.
